# Crossing the Septum: Step-by-Step Anatomy, Technique, and Enabling Technologies in Transseptal Puncture for Atrial Fibrillation and Electrophysiological Procedures

**DOI:** 10.3390/biomedicines14091952

**Published:** 2026-08-30

**Authors:** Dimitrios A. Vrachatis, Konstantinos A. Papathanasiou, Ioannis Anagnostopoulos, Maria S. Kousta, Sotiria G. Giotaki, Christos Piperis, Christos Karavasilis, Gerasimos Deftereos, George Marinos, Antonios A. Argyris, Konstantinos Raisakis, Andreas Kaoukis, Sotirios Patsilinakos, Georgios Giannopoulos, Gerasimos Siasos, Spyridon Deftereos

**Affiliations:** 1 Department of Biomedical Engineering, Medical School, National and Kapodistrian University of Athens, 115 28 Athens, Greece; 2 Electrophysiology and Pacing Department, Eugenideio Clinic, National and Kapodistrian University of Athens, 115 28 Athens, Greece; 3 Department of Cardiology, General Hospital of Athens “G. Gennimatas”, 115 28 Athens, Greece; 4 Cardiovascular Prevention & Research Unit, Clinic/Laboratory of Pathophysiology, Laiko Hospital, Medical School, National and Kapodistrian University of Athens, 115 28 Athens, Greece; 5 Third Department of Cardiology, Aristotle University of Thessaloniki, “Hippokration” General Hospital, 546 42 Thessaloniki, Greece; 6 Third Department of Cardiology, Medical School, National and Kapodistrian University of Athens, Sotiria Chest Disease Hospital, 115 27 Athens, Greece

**Keywords:** transseptal puncture, anatomy, step-by-step workflow, safety, troubleshooting

## Abstract

Transseptal puncture constitutes the foundational step required for left atrial access, serving as the critical gateway for pulmonary vein isolation, left-sided electrophysiological ablations, and percutaneous structural heart interventions. Although fluoroscopically guided transseptal puncture demonstrates high technical success rates alongside low reported complication rates, procedural training historically relies on unstructured apprenticeship models where overlooked mechanical nuances generate the majority of periprocedural adverse events. Methodological execution demands precise interatrial septal mapping, preprocedural left atrial thrombus screening, systemic anticoagulation, and rigorous hardware de-airing protocols. Real-time imaging using transesophageal or intracardiac echocardiography enhances fluoroscopic landmarks, facilitating precise localized puncture at the thin fossa ovalis while safeguarding adjacent anatomical structures such as the aortic root, posterior left atrial wall, and pericardial space. Modern auxiliary technologies, including radiofrequency crossing devices, provide systematic escalation strategies for fibrotic or altered septal substrates. Left atrial access must be unequivocally confirmed before dilator or sheath advancement using appropriate imaging, hemodynamic, or other procedural confirmation methods according to the technique employed. Ultimate procedural safety and operator competence depend on maintaining rigorous multi-modality confirmation protocols rather than relying on technical improvisation.

## 1. Introduction

Minimally invasive access to the left atrium has long been considered an “avaton”. Still, it has lately evolved as a “manual” skill on which a wide range of current interventional cardiology and electrophysiology rests. Pulmonary vein isolation for atrial fibrillation (AF), left atrial tachycardia ablation, ablation of left-sided accessory pathways, and a growing list of structural interventions all begin at the same place: a needle, a dilator, and a sheath crossing a few square millimeters of fossa ovalis [1,2,3]. Get that step right and everything downstream is possible; get it wrong, and an elective procedure may be terminated early or—even worse—a serious complication from the aorta or the pericardium shall arise.

Transseptal puncture (TSP) is therefore definitely worth teaching with extraordinary care, not as a trick passed hand to hand in the catheterization laboratory, but as a reasoned procedure whose every operative step has an anatomical and mechanical justification.

This review aims to be a detailed guide for the operator who has never performed a TSP, and for the more experienced reader who is rebuilding a transseptal program and wants the reasoning laid bare rather than assumed. Every effort has been made to meticulously delineate “clear” steps as carefully as the more challenging ones. Numerous TSP are routinely conducted in catheterization laboratories, resembling the continuous takeoffs and landings of airplanes at a major aviation hub. One shall not overlook that in such routine—still demanding—procedures the obvious steps are where most complications are actually born. The spine of the article is AF and electrophysiological (EP) practice; the expanding structural uses of TSP, left atrial appendage occlusion, mitral transcatheter edge-to-edge repair, and replacement are referred to only where they illuminate the core technique [3,4].

Fluoroscopy-guided TSP has been reported to be successful in 95–100% of cases across published series, with complication rates ranging from 0.0% to 6.7%, while mortality rates are reported roughly from 0.018 to 0.2% [3,5]. Simultaneous echocardiographic guidance is not mandatory for a safe and efficacious TSP. It is, though, a powerful adjunct, most valuable in difficult or failed cases. Undoubtedly, both fluoroscopy-only and echocardiographic-assisted tracks share the same anatomy, the same need for procedural confirmations, and the same safety principles. Herein, we aim to illuminate them so that an operator may confidently work in either. In this view, Table 1 provides a summary of the core terms.

## 2. Brief Historical Aspects

TSP procedure surprisingly does not have a long history, although it does not feel that way. Understanding its origins “rebrands” several of today’s “tricks” as legible inheritance rather than invention. Percutaneous transseptal left-heart catheterization was first described in the late 1950s as a diagnostic technique [6,7]. In 1959, John Ross Jr, working at the National Heart Institute, reported the founding method: a retractable needle passed through a cardiac (Cournand) catheter, the catheter tip placed in the right atrium against the fossa, the needle then advanced across the septum into the left atrium [6]. The idea itself came from a bedside observation by del Campo that a leg-introduced catheter slips readily through an atrial septal defect; if a defect could be crossed, so, deliberately, could an intact septum [6].

Ross’s experimental paper already contains much of what we still practice: needles fitted with *hub* indicator handles so the operator knows (assumes) where the tip points; the assembly directed posteriorly and somewhat medially; a change in resistance felt as the septum is crossed; and confirmation of position by blood sampling, pressure tracing, and contrast injection [6]. A companion clinical report by Ross, Braunwald, and Morrow the same year transported the technique into humans [8].

Two developments then shaped the tools we still name. The *Brockenbrough needle*, described with Braunwald and Ross in 1962, refined the curve and point into the design that remains the workhorse [9]. The *Mullins sheath* later standardized the long sheath-and-dilator system through which the needle now works [10].

A story that is worth remembering is that TSP nearly disappeared. After the advent of the flow-directed pulmonary artery catheter in the 1970s, the diagnostic need faded, and the procedure waned for almost a generation [7]. Its renaissance is entirely a creature of modern therapeutics: catheter ablation of AF above all, and more recently, the structural interventions that demand large-bore left atrial access [3,4,7].

Alongside transseptal access, other routes to the left heart were attempted and largely abandoned: the transbronchial, the posterior transthoracic, the direct apical (Brock), and the suprasternal (Radner) puncture, the last of which was used in substantial series into the 1960s before transseptal displaced it [11]. That history is not merely antiquarian: the venous, single-access, repeatable nature of TSP is exactly what stood the test of time.

## 3. Anatomy

Comprehension of the anatomical structures around which this deliberate technique is deployed is of utmost importance, first for safety and second for efficacy. Therefore, a comprehensive review of these landmarks establishes the baseline of this discussion and remains a recurring focus throughout the procedure.

### 3.1. The True Septum Is Small

What is assumed on fluoroscopy and appears on echocardiography like a broad interatrial “wall” is mostly not septum at all. The *true interatrial septum*, the tissue that can be crossed to arrive in the left atrium without entering extracardiac space, is only about 20% of the apparent septal area, essentially the floor of the fossa ovalis and its immediate margin [2]. The rest is infolded atrial wall, and in particular the posterior interatrial (Waterston’s) groove, where right and left atrial walls lie against one another with fat and pericardial space between [2]. A needle that crosses here has not entered the left atrium directly; it has tunneled into an extracardiac plane and subsequently to the left atrium. This is a mistake whose consequence may not declare itself until the sheath is removed at the end of the case (with the patient under effective and prominent anticoagulation; Section 11).

The safe target is the fossa ovalis: a thin, membranous, valve-derived floor set within a raised muscular rim, the limbus [2,3]. Embryologically, the fossa is the remnant of the septum primum, overlapped by the muscular septum secundum that forms the limbus; this is why the operator feels the tip drop over the limbus into the softer fossa rather than scrape across a uniform wall. In perhaps a quarter of hearts, the relationship of true to apparent septum is unusual enough to require the operators to adapt their tactical approach [2], which is the anatomical justification for never trusting a single view or a single landmark.

### 3.2. The Dangers as a Rose

**Figure 1:**
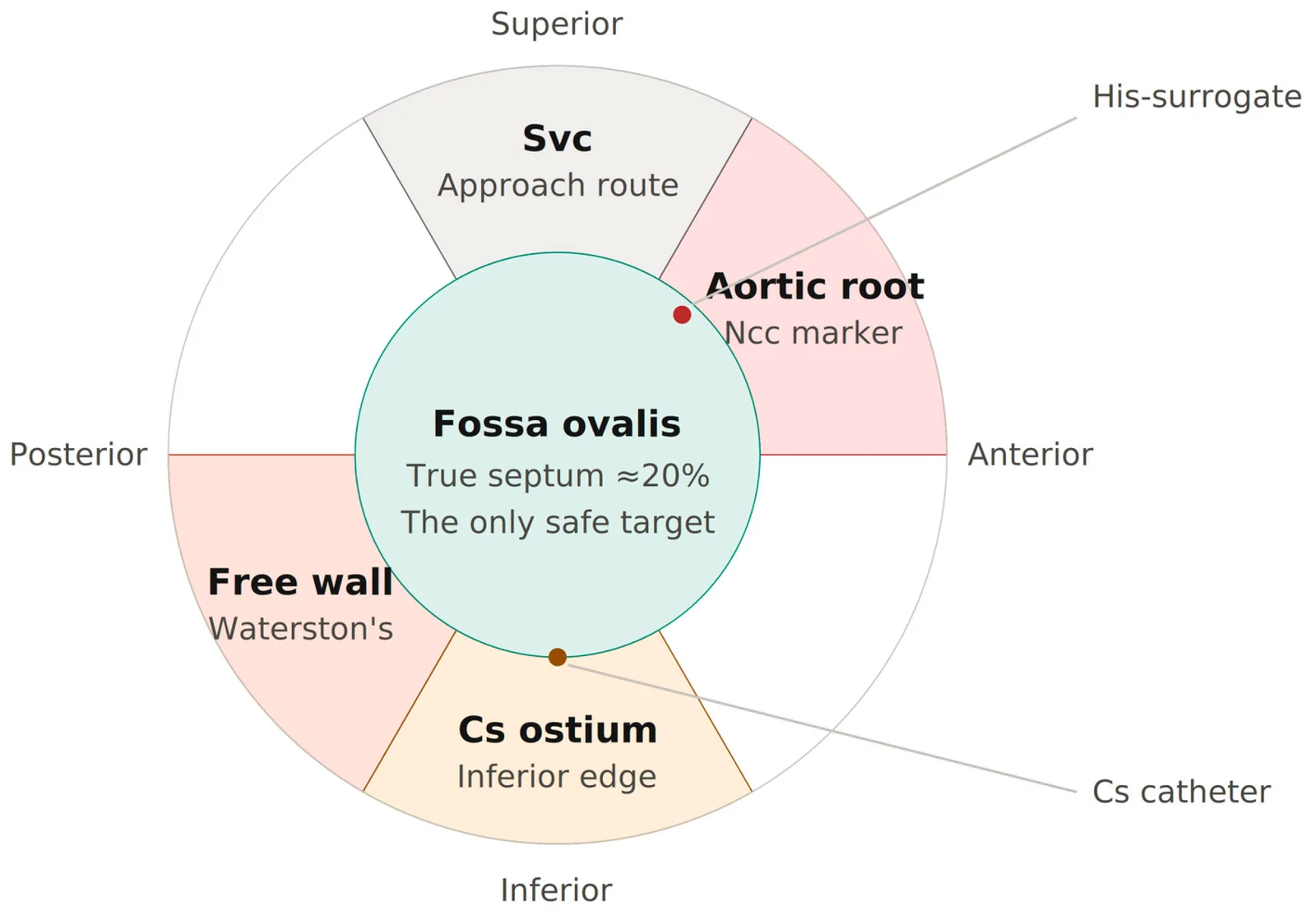
The “Danger Rose”. Hazards surrounding the fossa ovalis, arranged by direction. The septum is shown en face from the right atrium, superior uppermost, anterior to the reader’s right. The fossa ovalis at the center is the only tissue that leads to the left atrium. Each peripheral sector is a direction that punishes error. Anteriorly lies the aortic root, its non-coronary cusp marking the anterior boundary. Posteriorly lies the free wall and Waterston’s groove. Inferiorly lies the coronary sinus ostium at the lower septal border; superiorly, the assembly arises from the SVC, with the His region and aortic root converging superiorly and anteriorly. Filled markers denote the two catheters that fence the safe zone. CS, coronary sinus; NCC, non-coronary cusp; RAO, SVC, superior vena cava.

The fossa sits among structures that will punish inadvertence in characteristic directions. It is worth fixing them as a mental compass rose, the basis of the “Danger Rose” (Figure 1).

Anteriorly lies the aortic root; too anterior a puncture pierces it [1,3]. The *non-coronary cusp (NCC)* is its lowest point and therefore the most useful fluoroscopic marker of the anterior boundary, classically shown with a pigtail catheter in the aortic root, and in our center’s workflow marked instead by a retrograde diagnostic 0.035-inch guidewire, a low-cost equivalent [3,5]. Posteriorly lies the free wall and the Waterston’s groove; too posterior a puncture perforates the free wall or tracks extracardiac [2]. Inferiorly, the coronary sinus ostium marks the lower border of the septum; superiorly and anteriorly, the His region and the aortic root converge, and the His catheter conveniently sits in juxtaposition to the NCC [3]. Two catheters, one in the coronary sinus and one at the His position, thus may fence the safe zone from below/laterally and anteriorly, on which we lean throughout the fluoroscopic technique (see below; Section 7).

### 3.3. The Target Manipulations with the Procedure

**Figure 2:**
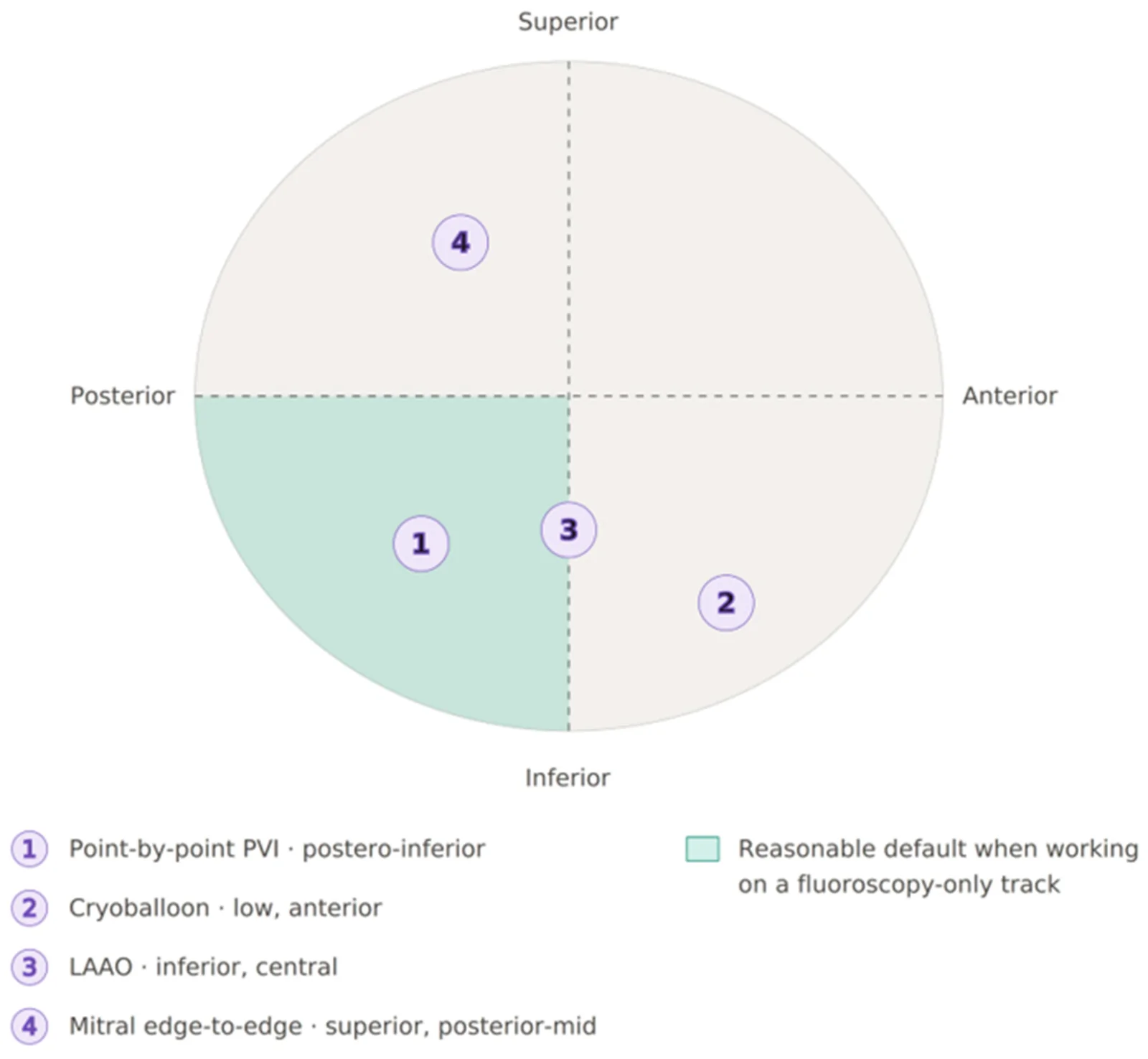
Site-specific puncture targets within the fossa ovalis. The fossa is shown en face from the right atrium, superior, uppermost, anterior to the reader’s right, and divided into four quadrants. Numbered markers indicate the preferred site for each: 1, point-by-point pulmonary vein isolation, best served postero-inferiorly; 2, cryoballoon ablation, where a low anterior position eases engagement of the right inferior pulmonary vein with the deflectable sheath; 3, left atrial appendage occlusion, favoring an inferior and central site; 4, mitral edge-to-edge repair, requiring a superior and posterior-mid position to gain the necessary height above the valve. LAAO, left atrial appendage occlusion; PVI, pulmonary vein isolation.

There is no single “correct” puncture point; the ideal site shifts with the procedure for which TSP is employed (Figure 2). For point-by-point PVI, a postero-inferior puncture serves best; for cryoballoon ablation, a low, anterior position eases engagement of the right inferior pulmonary vein with the deflectable sheath [3,7]. For left atrial appendage occlusion, an inferior/central site is preferred, and for mitral edge-to-edge repair, a superior and posterior-mid site gives the needed height above the valve [3,4]. Russo and colleagues formalized the fossa as four quadrants: superior/inferior × anterior/posterior [2]. Notably, only echocardiography can resolve the need for a specific puncture site (bicaval view for superior–inferior; short-axis view for anterior–posterior; four-chamber view for tenting-to-annulus distance) in a way fluoroscopy alone cannot [2]. Therefore, in the context of electrophysiology, when a fluoroscopy-only track is employed, aiming approximately for an “inferior and posterior” puncture would be reasonably efficacious to serve most procedures, allowing for flexible maneuvers of the catheters within left atrial structures.

### 3.4. Variant Septa the Operator Will Actually Meet

Expectedly, the septum is not always the thin membrane of the textbook, and the common variants each change the plan (see below; Section 10): the aneurysmal septum that tents deeply and springs the needle toward the far wall; the lipomatous or muscular-thick septum; and importantly and counter-intuitively the fibrotic “resistant” septum of the “repeat” procedure, which is usually not thick at all [12]. The same holds true for patients who have undergone cardiac surgical procedures, incorporating or not manipulations in the septum. Repeat, device-closed, and calcified septa likewise demand extra caution and should raise a red flag for employing the imaging track [3,7].

## 4. Before You Touch the Patient

Most of what keeps TSP safe happens before the needle is anywhere near the septum. This section walks the whole pre-puncture sequence, reminding that “an ounce of prevention is worth a pound of cure”.

### 4.1. Vascular Access and Tract Preparation

The right femoral vein is the most employed site of access for advancing the transseptal equipment and thereafter any required equipment for the index procedure. Left femoral veins may also be employed for TSP in the case the right side is not available. In our proposed workflow, the right femoral access is utilized for TSP only, and any other “supplementary” vascular access (for the coronary sinus and right ventricle catheters and intracardiac echocardiography, if employed) is directed to the left femoral vein. Of note, in many centers, one-sided femoral access only is employed. We do believe that this may lead to more friction and thus less maneuverability of the catheters and—potentially—more vascular access-related complications. Still, in a randomized study by Yu et al., unilateral groin puncture was associated with less access site pain, back pain, and total pain scores without significant differences in procedure time, major complication rate, and AF recurrence as compared with bilateral groin puncture [13].

Further, it need not be mentioned that ultrasound guidance for venous puncture should be incorporated into routine practice; a small discipline that reduces access complications and starts the case without any “non-significant” procedural burdens [2]. Recently, the ULYSSES trial demonstrated that ultrasound guidance for venous puncture is associated with fewer venous access site complications within 30 days of the AF ablation procedure, including arteriovenous fistula, false aneurysm, or access site bleeding requiring intervention or leading to prolonged hospitalization. Additionally, it reduced unintended arterial puncture and unsuccessful venous access attempts [14].

Historically, some operators place a small (4F, or now 2F) femoral arterial line for continuous blood-pressure monitoring, which doubles as the route for an aortic-root pigtail when that landmark is sought [3]; in our workflow and in view of prioritizing minimization of complications, we utilize right radial artery access.

As in most cases the eventual sheath is large (e.g., 13 Fr internal diameter/16.8 Fr outer diameter FARADRIVE™ Steerable Sheath, Boston Scientific), we advise that the vascular access site is accordingly prepared. This also, and importantly, facilitates effortless handling of the TSP equipment during retraction and manipulation to identify the final puncture site. Practically, this means preparing the vascular access site to about 14 Fr, starting by utilizing a blunt spread with a mosquito clamp, and serial up-sizing. The tactile feeling of the operator during initial preparation guides the final diameter that the access site should be expanded to. In the case that TSP equipment is advanced, and the operator feels that its steerability is limited, then it should be retracted and the vascular access site should be further prepared. Subsequently, short-length vascular access sheaths (or their dilators) can be gradually upscaled (i.e., 11–14 Fr) before transseptal sheath introduction to overcome subcutaneous tissue constraints, especially among patients with increased body mass index. Finally, after a successful TSP, the interatrial tract itself could be prepared utilizing the (long) dilator of the left atrial catheter that will be finally utilized. On the other hand, one could advocate against this extra exchange step as it could increase both procedure time and embolic events.

### 4.2. Imaging Strategy

The choice is not fluoroscopy or echocardiography but how much echocardiography, and for whom. Fluoroscopy alone is sufficient and safe in experienced hands across large series [3]; Intracardiac echocardiography and transesophageal echocardiography (ICE and TEE, respectively) provide direct septal imaging, site-specific targeting, and safety in difficult cases, at the cost of expense, complexity, and (for TEE) sedation [3]. ICE, used routinely in many centers, has additionally been associated with fewer bleeding complications and better anatomic orientation [15]. In our workflow, the default strategy is a fluoroscopy-led practice with echocardiography reserved for demanding cases.

### 4.3. Thrombus Screening and Contraindications

Existence of a left atrial or appendage thrombus is an absolute contraindication. In the case of definite proper anticoagulation, no further imaging is suggested by current guidelines to reassure it. However, preprocedural TEE or cardiac computed tomography within 48 h to exclude thrombus is the only definitive measure for this [12,15,16]. Such a strategy could especially be employed in patients with persistent AF, CHA2DS2-VASc score ≥ 3, hypertrophic cardiomyopathy, rheumatic heart disease, or cardiac amyloidosis since they are at increased risk of thromboembolic events [16]. Notably, ICE has recently been validated as an alternative to TEE for this purpose: in a multicentre randomized trial of 1810 patients undergoing AF ablation, ICE-based thrombus screening proved non-inferior to TEE, with periprocedural thromboembolic events occurring in 0.4% versus 0.6% of patients, respectively [17]. Preprocedural imaging timely reveals any foreseeable procedural peculiarities: the aneurysmal, thick, or previously repaired septum flagged on imaging changes the plan before the case starts. It may also define pulmonary vein anatomy and guide ablation strategy. Variants such as common trunks, a right middle pulmonary vein, roof-draining veins, or conjoined superior or inferior veins can influence the choice of ablation approach and modality [16].

### 4.4. Anticoagulation

The strategic point is now evidence-based: for AF ablation, uninterrupted therapeutic anticoagulation is safer than interrupting and bridging. In the randomized COMPARE trial, continuing warfarin at a therapeutic INR reduced periprocedural stroke/TIA compared with warfarin discontinuation and low-molecular-weight heparin bridging, and discontinuation emerged as a strong independent predictor of thromboembolism [15]. Uninterrupted vitamin-K antagonism (INR 2–3) is therefore the reference strategy [3,15]. Meta-analyses have shown that uninterrupted DOAC therapy reduces major bleeding by approximately 50–55% compared with uninterrupted warfarin during AF ablation [18,19]. Overall, the evidence supports uninterrupted anticoagulation with either DOACs or VKAs during AF ablation procedures.

Historically, intra-procedural heparin “structured” administration (exact dosage, timing relative to crossing the septum) and ACT targets (250–300 s, >300 s, 300–350 s, >350 s) vary per protocol/operator and are presented in Table 2. Yet, current guidelines support achieving and maintaining an ACT > 300 s during LA ablation [16]. Regarding timing of heparin administration, in our workflow, half of the desired dosage (100 units per kg) is administered immediately after successful vascular access, and the rest immediately after left atrial access.

### 4.5. Sedation, Positioning, Consent

Conscious sedation suffices for most fluoroscopy-guided EP work; general anesthesia is usual when TEE guides the puncture or for structural cases [3]. Cryoablation procedures are less painful compared with radiofrequency ablation, and conscious sedation has been proven safe and effective, with the additional benefit of shorter procedural times [20,21]. Regarding pulsed field ablation (PFA), the recently published COOPERATIVE-PFA trial demonstrated that a continuous remimazolam–ketamine-based deep analgosedation protocol is superior to an intermittent propofol–opioid boluses-based deep analgosedation or continuous propofol–opioid-based general anesthesia with secured airway due to a lower periprocedural risk of hypoxemia and hypotension [22]. Positioning is supine with defibrillation pads sited anteroposteriorly for AF cases [3]. Consent should name the specific serious hazards of this TSP tamponade, aortic puncture, thromboembolism at their real order of magnitude (major complications ~1–2%) rather than in vague terms [1,7].

### 4.6. The Pre-Flight Checklist

**Figure 3:**
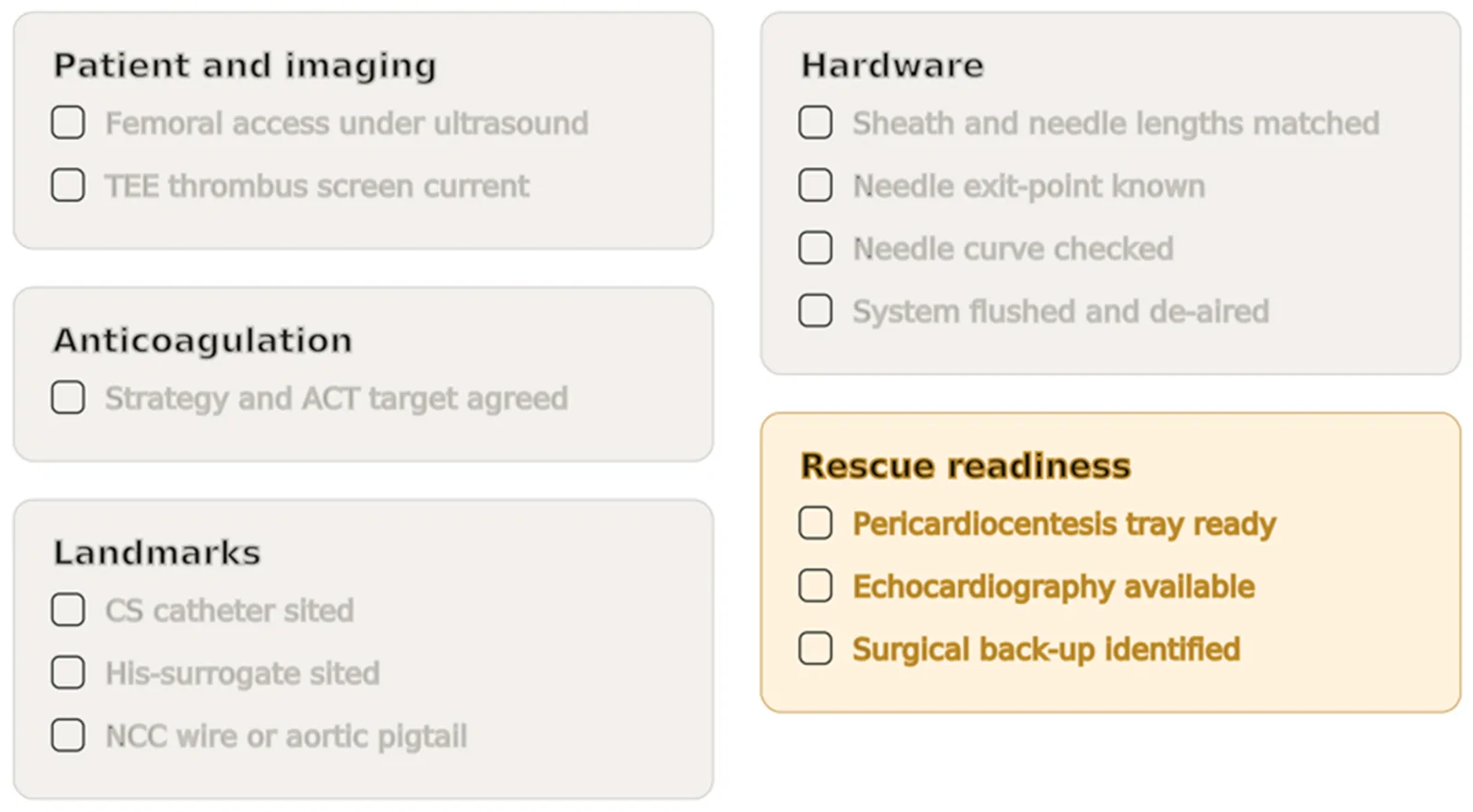
The pre-flight checklist. Items to be confirmed before the puncture assembly enters the patient, grouped by domain. Each box is a single verification answerable yes or no. ACT, activated clotting time; CS, coronary sinus; NCC, non-coronary cusp; TEE, transesophageal echocardiography.

Because the pre-puncture sequence is long and every item earns its place, Figure 3 sets out a checklist to run before each case. Of note, catheters or guidewires for anatomical landmarks and hardware might vary substantially among laboratories depending on operator experience and institutional protocols. We present a variety of safety measures utilized in our institution. Importantly, safety measures are complementary; demonstrating all of them concurrently is not always mandatory and mostly depends on operator discretion.

## 5. Hardware

The transseptal “system” is three nested tools: a needle inside a dilator inside a sheath, plus the guidewire that reaches the superior vena cava and the directional hub that indicates where the tip actually is. A surprising share of complications is prevented simply by understanding how these parts nest, and by preparing each one deliberately. A list of device families and representative brands is presented in Table 3.

### 5.1. The Needle

The workhorse is the Brockenbrough needle: a long, gently curved hollow needle with a directional hub. In conventional practice, it is a fixed-curve 18-gauge needle supplied in two curvatures: the BRK at roughly 19° and the BRK-1 at roughly 53° [7]. The larger BRK-1 curve suits larger or dilated atria [5]. The directional hub carries the flange (or arrow) marker that tells the operator where the tip is pointing, a feature present since the very first transseptal needles [5,6].

As packaged, the needle is often not curved enough for a given heart, and the operator may make an additional smooth secondary bend, typically 2–3 cm proximal to the primary curve [1], hand-shaping it [2] so the tip does not ride high toward the LA roof or the aortic root [3]. One caution is absolute: never bend the thin, tapered distal segment; fracture there can be catastrophic if it occurs inside the heart [3]. When the shape is wrong, it is safer to choose a needle with a different pre-formed curve than to overwork the tip [3].

### 5.2. The Dilator, the Sheath, and How They Nest

The needle sits within a dilator, which sits within a long sheath. Sheath families include the Mullins (e.g., 8F, 67 cm) [3]; fixed-curve types such as the Swartz SL0/SL1 [7]; steerable sheaths such as the Agilis [3] and the TorFlex, Preface, FastCath, and Channel sheaths [3,7] (Table 3). The discipline that matters is length and exit-point awareness: verify that the sheath and needle are matched in length and know exactly at what point the needle emerges from the dilator [3]. Advance the needle only to within a defined short distance of the dilator tip, variously taught as 2–5 mm proximal or within about 1 cm, keeping the tip unexposed until you intend to cross [7,12,23], and keep a thumb at the hub during pull-down so the sharp tip cannot slip out prematurely [3].

Preparation is simple and non-negotiable: flush the needle, dilator, and sheath with heparinized saline; place the needle through the dilator and sheath until the tip is visualized distally under fluoroscopy; and evacuate and flush the dilator contents before use [7].

### 5.3. The Stylet (Of the Needle)

A stylet is placed within the needle lumen during positioning to prevent the sharp needle from scraping or gouging the plastic dilator as the assembly is advanced [2,3]. Advance the needle with the stylet in until roughly 4 cm from the tip [2]; the stylet must not itself exit the dilator, or it can traumatize the SVC [3]. Our routine is: remove the stylet slowly and steadily while keeping the proximal end (operator end) below the level of the heart (to prevent air suction), immediately occlude the open hub with your thumb, “wet-to-wet” connection with the pressure line, then proceed [2]. The same thin stylet is also the tool for unclogging a needle whose lumen has occluded with clot [3].

### 5.4. The Guidewire and the Transducer

Access to the SVC is over a J-tipped guidewire (0.032–0.035″), advanced under fluoroscopy before the needle is exchanged [7,23]; note that some sheaths, among them Mullins, accept only a wire below 0.032″ [3]. The pressure transducer, which is to be connected to the needle tip (after removal of the stylet), is not an accessory in our workflow but one of the primary confirmatory means of position, as it monitors the tip throughout the pull-down and puncture [3,6] (Section 9).

## 6. De-Airing and Air Embolism

Air is the inaudible hazard of this procedure: it may not always produce a highly recognizable clinical picture the way a perforation does, and its consequences can masquerade as various entities depending on the destination of “embolic particles”.

The mechanism is worth picturing. An air bubble introduced through the transseptal system or during a catheter exchange can travel to the aortic root and lodge at the right coronary artery ostium, which sits anteriorly and superiorly; the result is inferior-territory ischemia with transient ST elevation [1]. It is usually self-limited but genuinely alarming intraoperatively. Intravenous fluids, high-rate atrial pacing, and vasopressors are all effective to mitigate transient episodes of hypotension. In severe cases of hemodynamic instability due to ischemia-related hypotension or arrhythmia, such as complete atrioventricular block or ventricular fibrillation, emergent coronary angiography is mandated, and securing radial artery access beforehand (as routinely employed in our workflow) might be lifesaving in these complicated cases [24].

The mimic matters as much as the mechanism. Not every ST elevation during TSP is air: some episodes are a Bezold–Jarisch-like vagal reflex or coronary vasospasm, and these may respond to atropine [1,3,25]. In one large cohort, transient ST elevation occurred in about 0.4% of cases without lasting sequelae [26]. The learning point is that in procedures incorporating TSP and LA access, catheterization laboratory members should actively track vital signs, recognize the pattern, and support the patient while remembering the opposite trap: hypotension during TSP is more often hemopericardium than vagotonia until proven otherwise (see below; Section 11) [3].

Herein we present our workflow’s de-airing routine. Before every puncture and before every flush, de-air, aspirate, and flush the needle and system [3]. Aspirate blood to clear any bubbles or thrombus [3]. With each catheter exchange through the long sheath, aspirate to remove clot, then flush, and keep the sheath continuously “perfused” with a slow-flow heparinized saline [3,15]. Every effort, at every stage, goes to avoid air intrusion [3,15].

## 7. The Fluoroscopy Workflow

Fluoroscopy workflow has long been utilized in the context of electrophysiology laboratories. The organizing idea in our workflow is simple: “cage” the fossa between reliable radiographic landmarks so that, even without echocardiography, the operator always knows (assumes) where the structures to be avoided lie.

### 7.1. The Landmark Set Building the Cage

The landmark set comprises four elements [3,5]: (a) a retrograde diagnostic 0.035” guidewire advanced to the non-coronary cusp a low-cost alternative to the aortic-root pigtail marking the anterior, aortic boundary at the aortic floor; (b) a multipolar catheter in the coronary sinus (CS), marking the inferior and lateral border of the septum (the CS ostium) and the course of the mitral annulus; (c) a catheter at the right-ventricular/His position used as a passive His-surrogate valued for its stable seat next to the NCC rather than for per-case His mapping; and d) the spine as a midline reference. Together, CS below and laterally, the His-surrogate and NCC above and anteriorly, and the spine at the midline, these fence the safe zone [3].

### 7.2. The Projections and What Each One Is for

Four fluoroscopic views are routinely used in our workflow, each answering one question. AP is the working view for the pull-down and the two “falls,” and the flange (direction-hub) orientation is read here. Right Anterior Oblique (RAO) 30° resolves anterior–posterior position, whether the tip lies anteriorly (toward the aorta) or posteriorly (behind the His/CS plane). The key distance to look for is that of the catheter from the spinal cord (keeping away points more anteriorly—i.e., toward the aorta—and shorter distance points more posteriorly—i.e., away from the aorta) [3]. Subsequently, a left lateral view is utilized to confirm remoteness from the aorta, with the tip sitting a defined fraction of the way from the aortic marker toward the spine [5]. Finally, LAO (about 45°, sometimes 30°) confirms proper orientation (and movement along with the multipolar CS catheter; about half to one vertebral body above the catheter) and is the puncture view [3,5].

### 7.3. The RAO Correction “Two Directions at Once”

**Figure 4:**
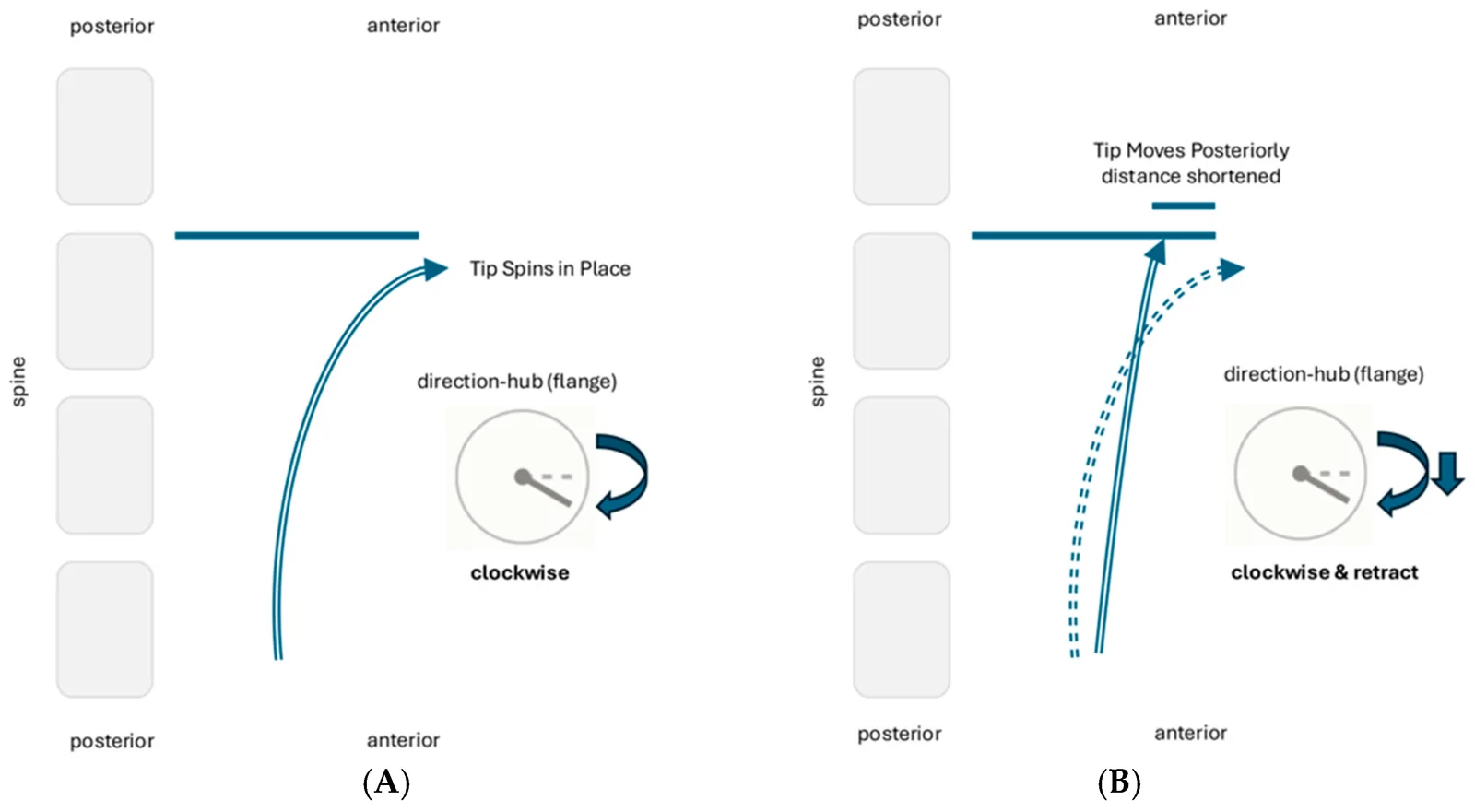
The RAO correction: two directions at once. (**A**) Rotation alone. (**B**) Retraction and rotation together. The horizontal reference line runs from the vertebral column to the needle tip; in this projection, its length is the measure of anterior–posterior position, greater length indicating a more anterior, aortic-facing tip and lesser length a more posterior one. Posterior lies to the reader’s left, anterior to the right. When the tip lies too anteriorly, the instinct is to rotate clockwise in place. Isolated rotation turns the needle about its own tip without altering the inclination of the shaft, so the tip does not relocate, and the distance from the spine is unchanged (**A**). The correction is to move in two directions at the same time: retract slightly and rotate slightly together. Retraction reduces the angulation of the shaft. In (**B**), the broken oblique outline is the starting position and the solid, more vertical outline the corrected one, and as the assembly straightens, the tip travels posteriorly along the same horizontal line, shortening the distance from the spine while preserving height. The direction-hub dial at the lower right of each panel shows the needle flange and the broken radius marks the starting orientation at about 3 o’clock, and the arrowed solid radius the corrected orientation at about 4 o’clock. RAO, right anterior oblique.

High-yield correction. When the tip is too anterior in RAO, the instinct is to rotate clockwise in place, but isolated rotation about a fixed contact point does not relocate the tip; it merely spins it around the same spot [2]. The fix, as described by Russo et al., is to move in two directions at the same time: retract slightly and rotate slightly together, so the contact point migrates to a safer, more posterior position [2]. Retraction reduces the inclination of the shaft, and as the assembly straightens, the tip travels posteriorly while preserving height (Figure 4). This is one of the single most useful things to teach a beginner.

### 7.4. The Canonical Sequence

**Figure 5:**
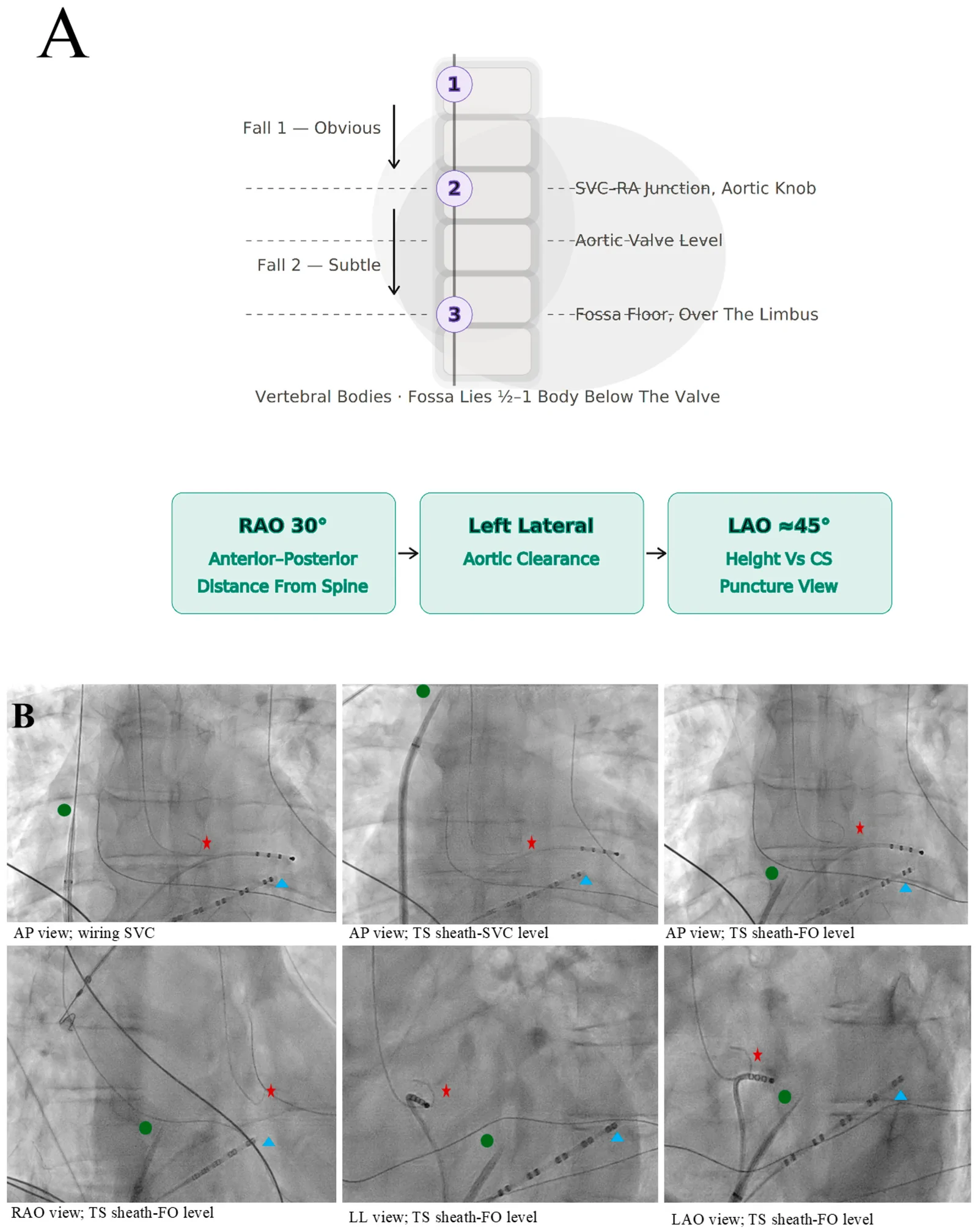
The canonical fluoroscopic sequence in our workflow: two falls, then three views. (**A**) Schematic. (**B**) Fluoroscopic stills (author’s own case). Upper register: the pull-down in AP. The assembled needle-in-dilator-in-sheath is retracted from the superior vena cava as a single unit (after stylet removal and connection of a pressure line) with thumb at the hub. Two falls are felt and seen. The first fall is obvious, as the assembly passes the aortic knob and the superior caval–right atrial junction. The second fall is subtle: the drop over the muscular limbus into the softer floor of the fossa. Measured from the SVC, the fossa lies roughly half to one vertebral body, approximately 1–3 cm, below the level of the aortic valve. Once landed, position is interrogated in a fixed order. RAO 30° resolves anterior–posterior position, read as the distance of the tip from the spine. Left lateral confirms clearance from the aorta, with the tip sitting a defined fraction of the way from the aortic marker toward the spine. LAO ≈ 45° confirms orientation and height against the CS catheter, about half to one vertebral body above it, and is the projection in which the puncture is made. AP, anteroposterior; CS, coronary sinus; FO, fossa; LAO, left anterior oblique; LL, left lateral; RA, right atrium; RAO, right anterior oblique; SVC, superior vena cava; TS, transseptal sheath. Red star: coronary wire inserted as a marker in aorta; blue triangle: CS catheter; green circle: TS sheath tip.

Here a step-by-step sequence for TSP is presented according to our suggested workflow (Figure 5):

1. Assemble at the SVC over the J-wire; exchange for the needle-in-dilator-in-sheath, stylet in, thumb at the hub. Remove the stylet and connect a pressure line.
2. Retract as a single unit in AP with the flange (direction-hub) at about 3 o’clock, watching for the two falls [1,3]: the first, obvious, as the assembly passes over the aortic knob and the SVC–RA junction; the second, subtle, as it drops over the limbus into the fossa; that second fall is the landing. From the SVC, the drop is classically about half to one vertebral body (≈1–3 cm) below the aortic-valve level [5].
3. Assess anterior–posterior in RAO: the tip should lie posterior to the His and parallel to the CS course [3].
4. Confirm clearance from the aorta in left lateral view [5].
5. Should modifications be required, the operator slightly retracts and rotates the system (rotate the needle flange toward 4–5 o’clock—i.e., clockwise—to attain a more posterior position or anticlockwise to attain a more anterior position). Reconfirm final position (RAO–Left Lateral) and puncture in LAO ~45° [2,5].

### 7.5. One-Handed Versus Two-Handed Retraction

**Figure 6:**
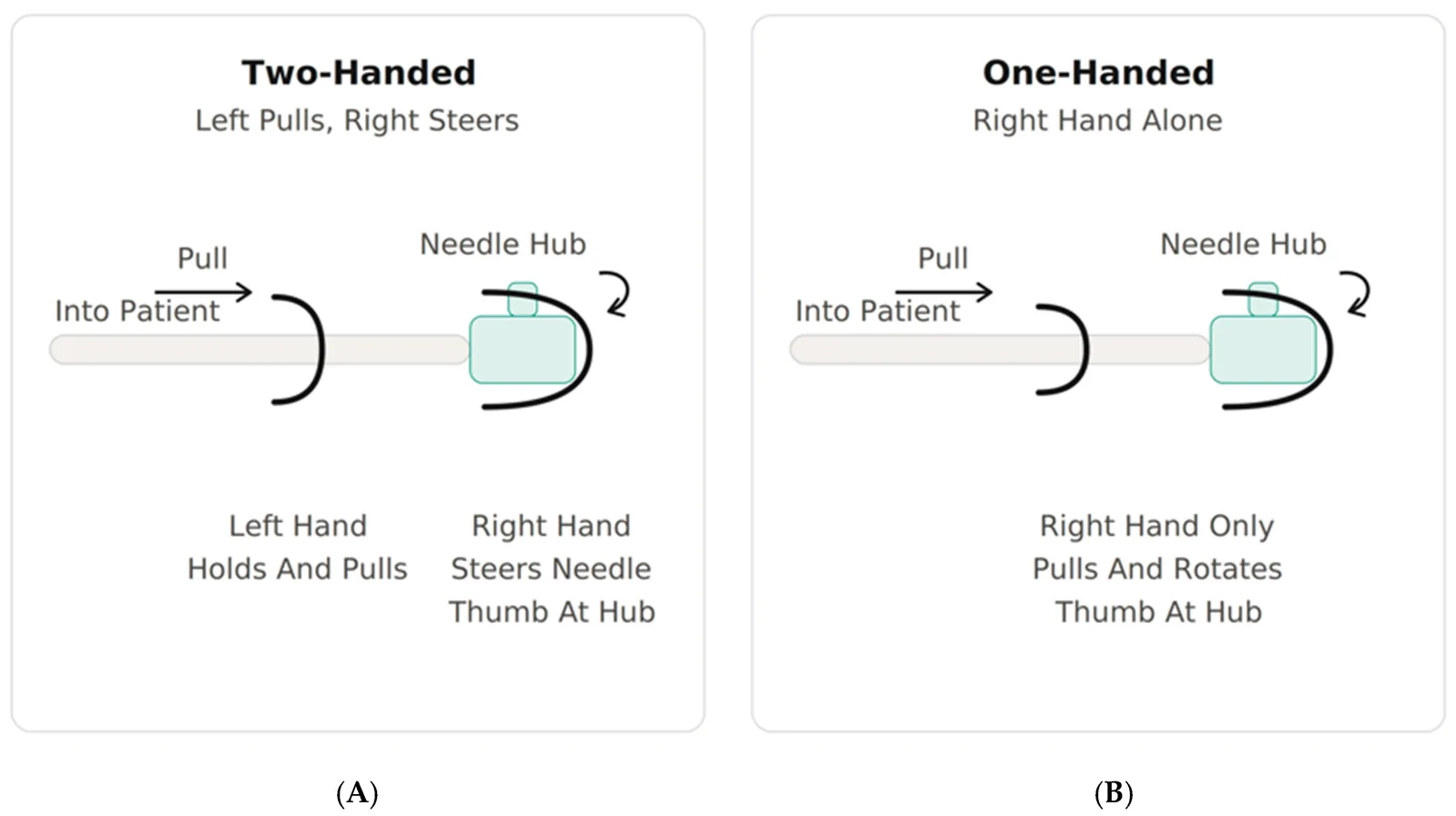
One-handed versus two-handed retraction. (**A**) Two-handed technique. (**B**) One-handed technique. The assembly is shown from the patient on the left to the operator on the right. In the two-handed technique, the sheath and dilator are held and pulled with the left hand while the needle is controlled with the right (**A**). In the one-handed technique, the whole assembly is retracted and rotated by finger control of the right hand alone (**B**). Brackets mark where each hand engages the assembly. In both, retraction is a withdrawal of the assembly as a single unit toward the operator, rotation is applied at the directional hub, and a thumb remains at the hub throughout so that the sharp tip cannot slip out of the dilator prematurely.

There are two reasonable ways to perform the pull-down (two-handed vs. one-handed). In the two-handed technique, the sheath/dilator is held and pulled with the left hand while the needle is controlled with the right. In the one-handed technique, the whole assembly is retracted and rotated with “finger” control of the right hand alone. (Figure 6) [2].

### 7.6. Tenting, Contrast, and the Pop

As the tip engages the fossa in the puncture view, it produces tenting, the pyramidal deformation of the membrane [2]. A small contrast injection (“staining”) against the septum may confirm engagement and outline the fossa, but is not integrated into our workflow [2,3]. Advancing then produces the pop, the sudden loss of resistance as the needle enters the left atrium; the same “change in resistance” Ross described in 1959 [6]. At this point, the operator should be very cautious: excessive tenting both makes the septum harder to cross and raises the perforation risk, while an aneurysmal septum can tent deeply toward the far wall [3]. The instant the septum yields, stop forward forces to the assembly promptly so that the latter does not lurch forward into the posterior wall [3,23] (see below; Section 11).

### 7.7. Advancing the Dilator and the Sheath

Exactly at this stage, nothing else advances until LA position is confirmed by the doctrine of multiple independent confirmations (see below; Section 9). Then the dilator, and then the sheath, are advanced over the needle into the left atrium [1,2]. As already described, the dilator itself can be advanced further across the septum to dilate the tract, easing the sheath’s passage [7]. Of note, echocardiographic guidance or electroanatomic mapping before TSP might substantially alter the doctrine of multiple independent confirmations, and as already stated, these safety measures are complementary and not obligatory in any institutional protocol.

### 7.8. Fluoroscopy-Only Failure Modes

Fluoroscopy alone cannot resolve puncture height or directly image the septum, and honesty about its blind spots is part of using it safely [3]. It cannot visualize the septum tenting in real time the way ICE can, so tactile and contrast cues shall carry more weight. It cannot reliably identify an aneurysmal or lipomatous septum in advance. Two-sheath “probing” and giant-atria variants that call for increased needle curvature or a BRK-1 are fluoroscopic workarounds for difficult anatomy [5]. The very posterior “stitched walls” of the right and left atria are a specific fluoroscopic hazard: a puncture there can track between the apposed walls of Waterston’s groove rather than into the LA [2,5] (see below; Section 11). When fluoroscopy leaves genuine doubt, the correct step is never to push harder but to add echocardiography, electroanatomic mapping, or to withdraw and restart [3].

## 8. The Echocardiographic Workflow (TEE and ICE)

Echocardiography does not replace the anatomy or the confirmation of the fluoroscopic track; it adds a direct way of observing the septum, and crucially it resolves puncture height, which fluoroscopy cannot. Importantly, a zero-fluoroscopy strategy that combines ICE with electroanatomic mapping can reduce radiation exposure and obviate the need for additional vascular access otherwise used for fluoroscopic anatomic landmarks, particularly in workflows supporting less experienced operators as described previously.

### 8.1. The TEE Views and What Each Resolves

Three TEE views do most of the work, and it helps the novice to learn each as the answer to one geometric question [2]. The *bicaval view* resolves the superior–inferior axis: how the tip sits on the septum with regard to the axis delineated between the superior and inferior vena cava. The *short-axis (aortic-valve) view* resolves the anterior–posterior axis: how close the tip is to the aorta in front or the free wall behind. The *four-chamber view* resolves height in the other sense that matters: the tenting-to-annulus distance, i.e., how far above the mitral valve the puncture will sit (low vs. high) [2]. Read together, these give a three-dimensional fix on a target that fluoroscopy can only place in two dimensions.

### 8.2. The ICE Home View and Low or Zero Fluoroscopy Approach

Intracardiac echocardiography images the septum from within the right atrium and is, in some centers, the routine default. From a stable home view, the operator monitors the sheath/needle contact and tents the fossa in real time; the crossing itself is often marked by a small burst of microbubbles appearing in the left atrium as the tip passes through [7,23]. ICE demands its own venous access and adds catheter cost, but it maintains imaging under the operator’s own control and has additionally been associated with fewer bleeding complications, better anatomic orientation, and easier confirmation of catheter contact [4,15].

Fluoroscopy alone may not always ensure procedural safety in a solid manner. ICE (and, as already mentioned, TEE) addresses this limitation by providing direct, real-time visualization of the fossa ovalis and adjacent structures. It helps operators recognize anatomic variants, confirm tissue contact by observing septal tenting before puncture, and use complementary short-axis (anterior–posterior) and long-axis (cranio-caudal) views to precisely localize the needle. Together, these perpendicular views enable site-specific transseptal crossing, such as a central-inferior puncture for pulmonary vein isolation, a posterior-inferior site for left atrial appendage closure, or an anterior-inferior approach for antegrade left ventricular access. A patent foramen ovale may also be identified, which may occasionally permit direct left atrial access without formal puncture [27].

A key advantage of ICE-guided TSP is the ability to perform the procedure with little or no fluoroscopy (without requiring any personnel except for the interventionalists). This fluoroless approach relies on stepwise direct visualization: advancing the guidewire into the superior vena cava, passing the sheath over the wire, and carefully drawing the sheath–needle assembly toward the fossa before positioning it at the intended puncture site. Successful crossing is confirmed by observing the needle traverse the fossa, with flattening of the previously tented septum, and can be further supported by saline or contrast injection showing microbubbles entering the left atrium. ICE also reduces the need for repeated contrast injections to confirm sheath or needle position, fossa engagement, and left atrial access, which is especially useful in patients with renal impairment or contraindications to contrast [27].

A proposed zero-fluoroscopy workflow for AF catheter ablation combines 2D ICE, 3D electroanatomical mapping, and pre-acquired computed tomography or magnetic resonance imaging models. The technique centers on three right atrial steps: ICE-guided cannulation of the coronary sinus with a navigation-enabled catheter, real-time ICE tracking of guidewire and transseptal sheath advancement into the superior vena cava, and direct visualization of fossa ovalis engagement and septal tenting during precise left atrial puncture. Marking the transseptal sheath course on the mapping system can also support rapid fluoroless re-entry if access is lost [28]. This zero-fluoroscopy TSP strategy also eliminates the need for conventional anatomic landmarks, such as an NCC guidewire or CS catheter, and can be performed without additional vascular access.

The clinical value of ICE is greatest in anatomically challenging septa. In aneurysmal septa, ICE shows excessive tenting and the limited distance to the opposing atrial wall, highlighting perforation risk and supporting gentle, controlled needle advancement, sometimes with a radiofrequency needle or dedicated TSP system. In fibrotic or thickened fossae, increased echogenicity signals greater resistance and a higher risk of perforation. Lipomatous hypertrophy creates the typical hourglass septal configuration around an otherwise normally thick fossa; in this setting, ICE confirms that the sheath drops into the narrow target rather than the surrounding hypertrophied tissue. By directly displaying these variants, ICE allows operators to adjust manual force, judge the distance from the tented fossa to the opposite wall, and determine in advance whether specialized crossing tools are needed, decisions that are much less reliable with fluoroscopy alone. ICE is equally valuable when the septum has been surgically or percutaneously modified. In the presence of a surgical transseptal patch, ICE directly images patch thickness and the surrounding native tissue, since crossing the patch typically requires more force and carries an increased risk of inadvertent left atrial wall perforation, again potentially warranting a radiofrequency needle [27].

When an atrial septal occlusion device is present, ICE delineates the device and any residual native septal rim, most often inferoposteriorly, allowing the sheath–needle assembly to be positioned in an accessible target. If no native rim is available, ICE-guided puncture through the device with a radiofrequency needle may be necessary. A related but distinct setting is transbaffle puncture after Senning or Mustard repair, in which a surgically created atrial baffle redirects venous inflow and often divides the cavotricuspid isthmus. When atrial tachycardias arise in the systemic atrium or involve the isthmus, ICE is essential for identifying the baffle and selecting a safe puncture site [27].

Across these complex scenarios, ICE shifts TSP from “estimation” (relying on fluoroscopic landmarks and tactile feedback) toward “direct anatomic visualization”. This can reduce procedural risk and enable early recognition of complications, such as pericardial effusion or thrombus formation, allowing timely intervention [29].

A recent single-center study evaluated a standardized fluoroless workflow combining electroanatomic mapping with left atrial ICE and compared pulsed field ablation (PFA) with radiofrequency ablation (RFA). PFA was associated with greater procedural efficiency and better short-term rhythm control in paroxysmal AF, although larger multicenter randomized studies are needed to confirm these findings [30].

### 8.3. A Shared Vocabulary and Combined Choreography

The two tracks are not rivals: the landmarks and confirmations are the same, and echocardiography simply makes some of them visible. Tenting is tenting whether inferred from contrast on fluoroscopy or seen directly on ICE; the fossa is the fossa. In a combined workflow, the operator might use fluoroscopy for the pull-down and the two falls, then turn to echo to confirm height and anterior–posterior position before committing to the puncture, and back to the shared confirmations (see below) before advancing the critical parts of the equipment beyond the septum. Importantly, many high-volume laboratories with experienced operators perform zero fluoroscopy procedures and, in these cases, TSP is mainly guided by electroanatomic mapping and echocardiography.

### 8.4. Choosing a Modality: An Objective Comparison

In our institutional protocol, we follow fluoroscopy-led practice for routine cases, with echocardiography brought in for site-specific structural targets, for difficult or previously failed to cross septa, and where an inexperienced operator benefits from direct confirmation. One series found that TEE corrected a puncture position that fluoroscopy alone had placed wrongly in about one every six to seven cases [3]. The point is capability in both, not allegiance to one. Table 4 compares the capabilities of each track.

## 9. Am I in the Left Atrium?” the Doctrine of Confirmation

**Figure 7:**
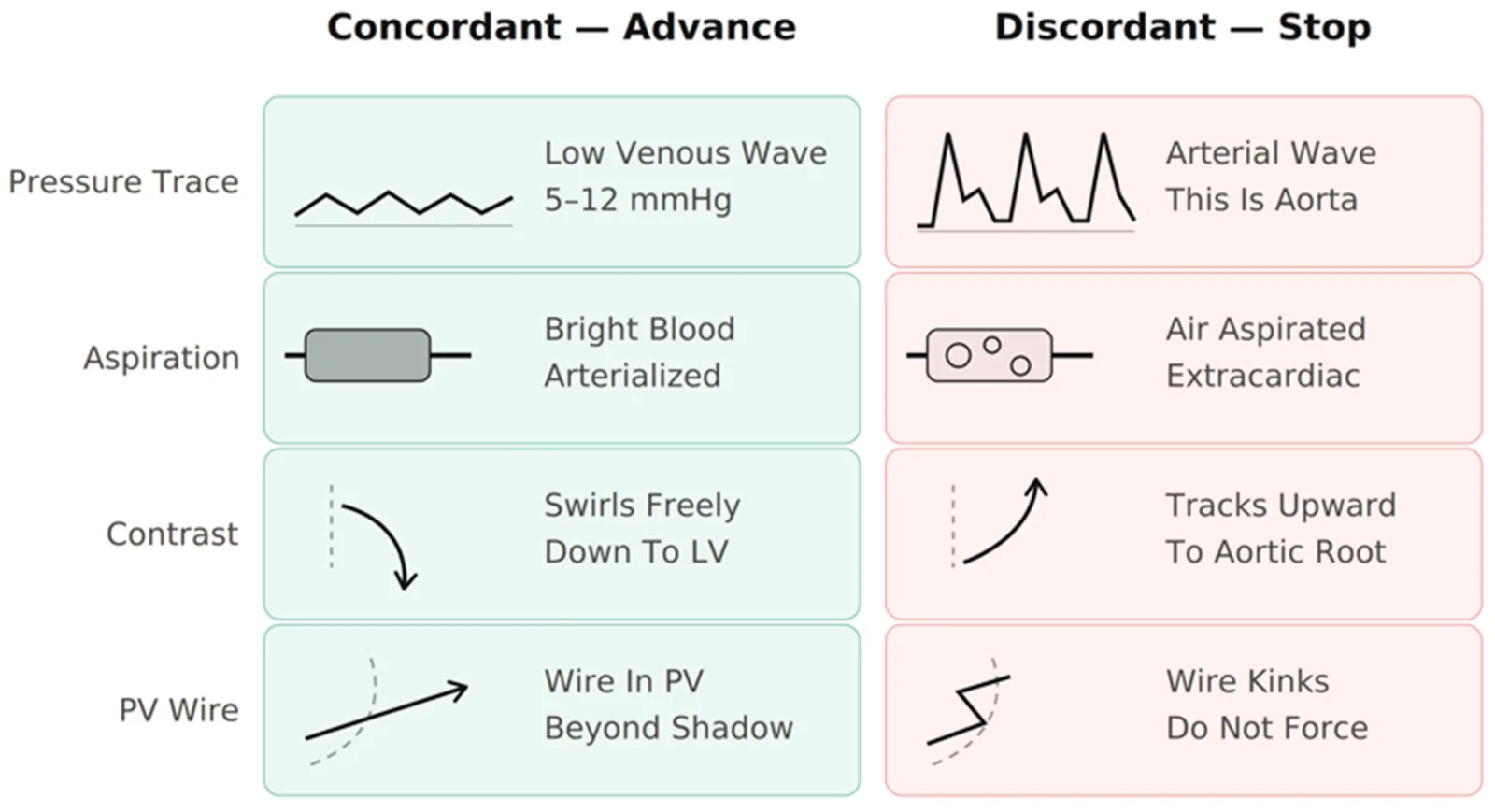
LA position confirmation maneuvers in our workflow. Before advancing the dilator or sheath, position should be verified using multiple independent confirmations. Each row shows a confirmation method alongside concordant and discordant findings. LA, left atrium; LV, left ventricle; PV, pulmonary vein; RA, right atrium.

No step in this procedure is more important, and none is more often hurried. Before the dilator or sheath advances a single millimeter, the operator must know, not hope, that the needle tip is in the left atrium. Contemporary workflows differ substantially. Under direct ICE or TEE visualization, for example, transseptal crossing can be directly visualized, and RF-wire-based or zero-fluoroscopy procedures may use different confirmation strategies. The safe practice in our workflow is a doctrine of multiple independent confirmations: no single sign is trusted alone, and agreement across all is required before anything crosses (Figure 7) [2]. This doctrine is old enough to be in Ross’s founding paper, which already confirmed position by blood, pressure, and contrast together [6].

### 9.1. Pressure Waveform

Connect the transducer to the TSP needle (if not routinely done earlier) and read the trace. A left-atrial waveform (a low, venous-type pressure, roughly 5–12 mmHg) replacing the right-atrial trace is a primary confirmation [3]. The dangerous positive is an *arterial* waveform, that is, the aorta, and the rule is: unconditionally do not advance the dilator or sheath, withdraw the needle, and watch the patient closely for tamponade [3]. Pressure may also distinguish LA from pulmonary artery tracings if the tip has taken an unexpected course.

### 9.2. Blood Aspiration Color, Saturation, and the Air Rule

Just after exposing the needle from the sheath and the pressure waveform is inspected, the pressure transducer is disconnected from the needle. The next step is to aspirate via the needle. Bright, arterialized blood suggests the left side; dark blood the right, a distinction whose antecedents reach back to the suprasternal era [3,11], and an oxygen-saturation sample settles genuine doubt [3]. One aspiration finding overrides all reassurance: aspirating air means you are not in the left atrium at all. Should bubbles of air be aspirated in the syringe, immediate reassessment is required, and advancement of the sheaths is to be avoided. Air may originate from an imperfect connection between the syringe, needle, or other components of the system, whereas true extracardiac advancement into an air-containing structure may involve structures such as the bronchial tree or esophagus. In either case, aspiration of air should prompt immediate reassessment and should not be interpreted as a specific marker of a particular anatomical location.

### 9.3. Contrast Injection

A small contrast injection through the needle completes the set. The syringe that is connected to the TSP needle to aspirate is prefilled with a few milliliters of contrast dye. If aspiration provides the expected arterialized blood, then a gentle contrast push is conducted in the left atrium. The dye swirls freely, and its initial course runs downward toward the left ventricle, not upward toward the aorta [2,3]. Contrast that stains and holds in septal tissue indicates the tip is still in the septum (not yet across) and usefully marks the fossa for a repeat attempt [12]. Contrast heading up toward the aortic root is the signature of an anterior “mistake”. Importantly, during TSP, contrast injection provides immediate feedback on needle position and helps prevent tissue injury or extracardiac perforation. When the needle is engaged in the dense muscular limbus or partially embedded in the atrial rim, injection resistance is high, and fluoroscopy shows fixed, non-dispersing contrast staining; this persistent ‘smear’ or ‘tattoo’ suggests intramural positioning and should prompt slight withdrawal toward the central fossa. If the assembly enters Waterston’s groove, contrast may pool or track slowly along the interatrial sulcus without washing out, indicating an extracardiac course and a risk of tamponade if the system is advanced. In contrast, when the needle crosses the thin fossa ovalis into the left atrium, contrast injection is low-resistance and produces a rapidly dispersing cloud that washes downstream with normal flow, confirming appropriate positioning before sheath advancement.

### 9.4. Pulmonary Vein Wiring

Subsequently advancing a fine (0.014″) coronary/angioplasty wire through the needle into a pulmonary vein (in most cases into the left superior vein) is a strong confirmation: the wire runs out beyond the cardiac silhouette, outside the heart shadow, where no right-sided structure would let it go [3]. The difficult-case branch: when the wire will not enter a pulmonary vein, do not force it and do not treat failure as disproof. Proximal kinking of the wire may mean the tip is in the right-ventricular outflow tract or the left pulmonary artery rather than the LA [3]; the correct response is to fall back on the other confirmations (pressure, aspiration, contrast) and, if doubt remains, withdraw and reconfirm position rather than push.

### 9.5. Putting Together the Confirmation Algorithm

**Figure 8:**
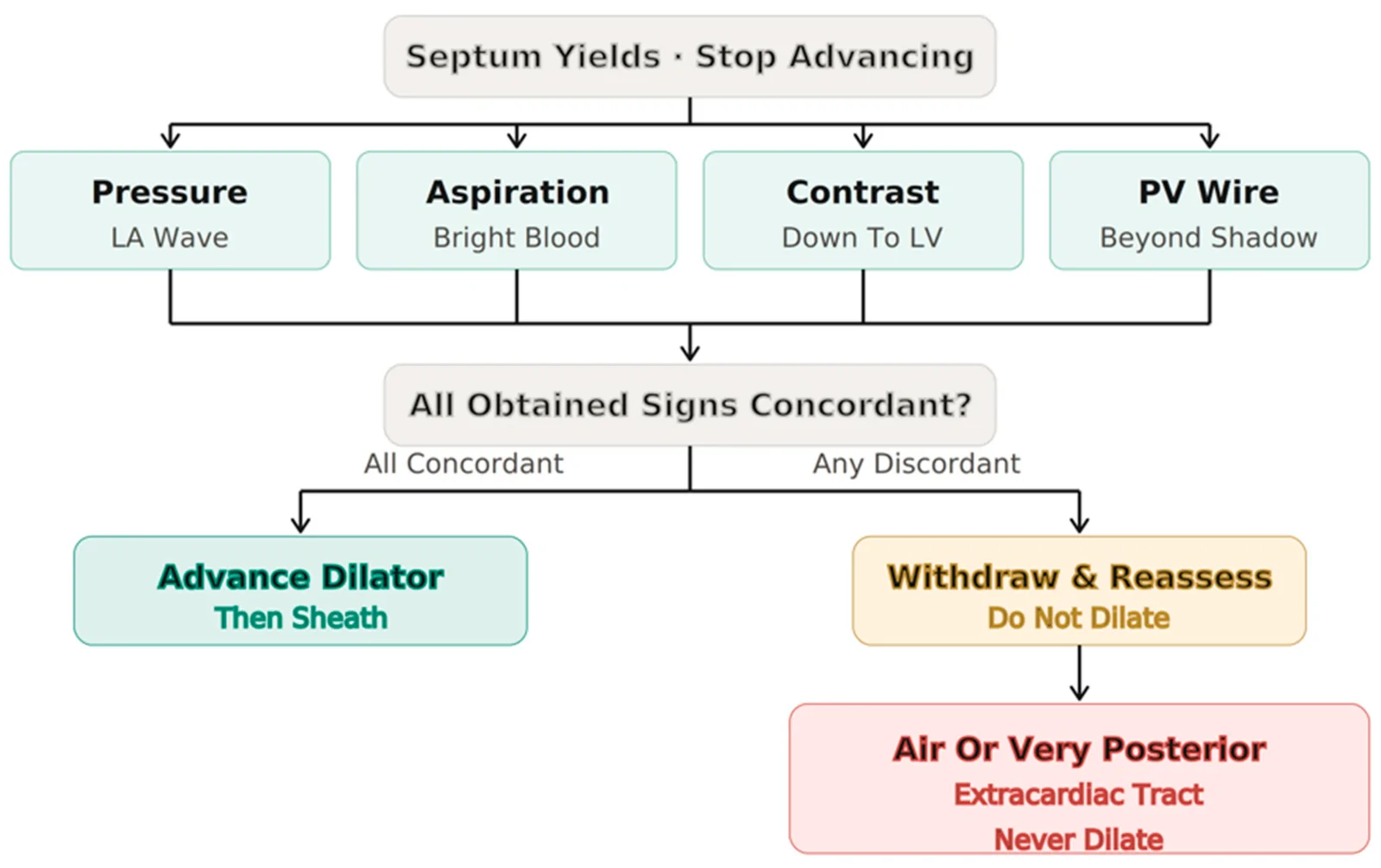
The LA position confirmation algorithm. Four signs are obtained: a left-atrial pressure waveform, aspiration of bright arterialized blood, contrast swirling downward toward the left ventricle, and a fine wire passed into a pulmonary vein and advancing beyond the cardiac silhouette. Concordance across the signs obtained licenses advancement of the dilator and then the sheath. LA, left atrium; LV, left ventricle; PV, pulmonary vein.

The confirmations are combined, not ranked. In practice: obtain an LA pressure trace; aspirate and check color (and saturation if unsure); inject a little contrast and watch its direction; and, when needed or for extra certainty, pass the PV wire (especially if working on a fluoroscopy-only track). Concordant signs: LA pressure waveform, arterialized blood, downward-swirling contrast, a wire in the pulmonary vein, and advancing the dilator. Any discordant sign an arterial trace, aspirated air, contrast to the aorta, or a wire that kinks proximally stops the procedure: withdraw, reassess, and only then decide whether to re-puncture. This overlapping logic is the single most protective habit in transseptal work (Figure 8) [2,3].

## 10. Challenging Septa and Enabling Technology

Most transseptal punctures are uneventful; the ones that do not tend to fail in recognizable patterns, and each pattern has a tailored response. The unifying rule is that difficulty never licenses force: every escalation below is still gated by the confirmation doctrine and the never-dilate creed.

### 10.1. The Septa the Operator Will Meet

The *aneurysmal* septum tents deeply and can spring the tip toward the far wall as it finally provides a setup for posterior perforation [3]. The *thick, fibrotic, or scarred* septum of the repeat procedure is the commonest “resistant” type, and its lesson is counter-intuitive: in one series such septa were consistently under 2 mm thick (i.e., normal), the resistance reflecting fibrosis from prior transseptal access rather than hypertrophy, with the number of previous punctures the only predictor [12]; about 5% of punctures in that consecutive series proved resistant to the conventional needle [12]. The lipomatous or muscular septum simply demands more than the usual force to cross. The giant or rotated atrium distorts the usual geometry and may call for increased needle curvature, a BRK-1, or two-sheath probing [5]. And the device-closed or patched septum is a special case in which imaging (ICE/TEE, sometimes CT) should be considered mandatory to find a safe window before any needle moves [3].

### 10.2. The Escalation Ladder

When a correctly positioned needle will not cross, escalate in a single, disciplined sequence rather than reaching straight for force. The ladder is a teaching synthesis; every step is gated by confirmed fossa position and the never-dilate creed:

- Optimize the basics first: reconfirm the tip is truly in the fossa; reshape the needle with a secondary bend; consider a deep-inspiration maneuver, which pushes the septum rightward to aid puncture but only when there is no doubt about needle position [3].
- Advance a stylet through the needle to concentrate force at the tip [1].
- Cross with a wire: a sharp-tipped nitinol wire (SafeSept) passed through the needle, which resumes an atraumatic J-shape on entering the LA [1,3], or the stiff back-end of a 0.014″ coronary wire [2].
- Apply radiofrequency: a dedicated RF needle (NRG, blunt closed tip) that crosses with minimal force and less “jump” [7], or, where a dedicated needle is unavailable, improvised RF with a standard ablation catheter touched to the needle hub [12] or electrocautery applied to the needle [23]. Watch for RF overshoot [2] and for tissue coring/plugging of the needle, an embolic hazard [3]. Two safety notes on the RF rung. With electrocautery, initiate energy before the tip leaves the dilator and keep the needle sheathed until it fires; this minimizes the power needed and the forward lurch [23]. RF removes the need for force, which is precisely why it reduces the sudden give that drives posterior-wall perforation [7,23], but it does nothing to excuse an unconfirmed position.

### 10.3. Enabling Technology Beyond the Needle

Several technologies change the difficulty curve rather than the crossing itself. SafeSept™ Transseptal Guidewire (Pressure-Products Inc., San Pedro, CA, USA) is a floppy “J-tip” needle wire system (120 cm nitinol guidewire with 0.014-inch diameter) that can facilitate safe left atrial access in challenging cases such as redo procedures, resistant septum and septal aneurysms [31,32,33]. A stiff-body pigtail or RF exchange wire (ProTrack; VersaCross, Boston Scientific) both prevent left-atrial overshoot of a large sheath and serve as a rail along which the tract is dilated and the big sheath delivered [4,7]. Dedicated steerable sheaths such as the VersaCross steerable system (combined with RF wire for transseptal puncture) have been associated with reduced time to transseptal puncture compared with standard workflow involving sheath exchange [34].

Further, in the PFA pulmonary vein isolation era, accumulating evidence supports fluoroscopy-guided direct Faradrive (Boston Scientific) sheath access for transseptal puncture. Of note, it has been associated with shorter procedure and fluoroscopy times, lower procedural costs, and no apparent compromise in acute procedural safety or success. These findings support simplifying the workflow by eliminating sheath exchange when performed by experienced operators [35,36]. More recently, the Faradrive sheath (Boston Scientific) has been combined with an RF guidewire (VersaCross, Boston Scientific) in a simplified direct-access strategy that avoids both a rigid metal needle and over-the-wire sheath exchange, thereby reducing procedural complexity, total procedure time, and fluoroscopy exposure [37,38].

In non-fluoroscopic AF ablation, randomized evidence suggests that ICE-guided transseptal puncture with an electrified J-wire is as safe as conventional mechanical-needle puncture and may be more efficient. In detail, the electrocautery generator is set to electrocoagulation mode with power at 20 W. The cautery pen tip is placed in contact with the proximal portion of the guidewire, and energy is delivered to facilitate left atrial access under direct ICE view. If microbubbles appear at the tenting site, the energy time can be reduced to <2 s [39]. Chen et al. further reported that predefined electrophysiology-based criteria may allow safe and successful zero-fluoroscopy transseptal puncture guided by 3D electroanatomic mapping. Patients meeting all four criteria were considered suitable for 3D-guided transseptal puncture, whereas those meeting none required ICE guidance. The major criterion was a sudden disappearance or marked reduction in the unipolar current-of-injury amplitude during withdrawal of the transseptal needle from the muscular limbus to the fossa ovalis, enabling precise localization of the fossa. Three minor criteria reduced atrial unipolar or bipolar potential and visible fossa ovalis protrusion provided additional procedural support [40].

Nevertheless, less may sometimes be more. In a large database spanning more than a decade and including over 4000 patients, Letsas et al. reported that a modified needle-free rotational probing technique achieved left atrial access in 72% of cases without compromising safety. In this approach, the mechanical needle tip is advanced to the dilator tip to support the transseptal system. Once the assembly is properly positioned against the septum, it is gently rotated between the 4 and 6 o’clock positions under controlled pressure for up to 2 min. Successful entry is marked by a sudden “jump” into the left atrium and confirmed by advancing a coronary guidewire into the left pulmonary veins [41]. Enabling technologies are summarized in Table 5. 

## 11. Complications

Overall, transseptal puncture is safe, but individual errors can be unforgiving. In large series, major complications occur in about 1–2% of cases [1,7], overall complications in roughly 0.74–1.3%, rising to approximately 2.8% in smaller series, and mortality is reported at about 0.05–0.1% [3,14]. These figures are reassuring only when each specific hazard is understood and actively rehearsed.

### 11.1. Cardiac Tamponade

Tamponade is the dominant serious complication and the one every operator must be ready to manage in minutes, not hours. It arises from perforation posterior to the fossa (the free wall) or anterior to it (the aortic root), or from the assembly lurching forward as the septum finally gives [3,23]. Reported tamponade risk is 0.4–1.3% for PVI [16]. Management is immediate cardiac ultrasound confirmation, pericardiocentesis with the tray already open, heparin reversal with protamine (and fresh-frozen plasma or factor concentrates if the patient is on warfarin), and surgical backup [3,15,24,42]. The governing reflex: hypotension during TSP is hemopericardium until proven otherwise, never lightly attributed to vagotonia [3].

### 11.2. Aortic Puncture and the Never-Dilate Rule

Inadvertent aortic puncture is uncommon [2], but outcome depends on the immediate response. If only the needle enters the aorta, it can usually be withdrawn with close monitoring for hemopericardium [3]. If the dilator or sheath has entered the aorta, the management changes: do not withdraw it. Leave it in place, consult cardiothoracic surgery, and prepare for controlled repair; in selected cases, removal should occur only with surgical support present [3]. This is the essence of the never-dilate rule: enlarging an unconfirmed tract can turn a needle puncture into a catastrophic injury [2,3].

### 11.3. Waterston’s Groove Delayed Tamponade

The most dangerous complication may be the one that remains initially silent. A very posterior puncture can miss the true septum and enter Waterston’s groove, the extracardiac plane between the apposed atrial walls, or “stitched walls,” seen fluoroscopically [2,5]. In this location, confirmations may be equivocal, and aspiration may not yield air. Because the sheath can temporarily tamponade the extracardiac tract, bleeding may appear only after sheath removal at the end of the procedure, causing delayed tamponade in recovery [2]. Prevention is upstream: respect the anterior–posterior views, follow the no air rule (described above), and never dilate a posterior tract unless left atrial entry is unequivocally confirmed.

### 11.4. Embolism: Air and Thrombus

Air embolism and its coronary/vagal presentation have already been described in the present article. Thromboembolism is the other arm: reported stroke/TIA after AF ablation ranges historically from about 0.9% to 5% depending on anticoagulation strategy [15]. Contemporary studies report lower risk for stroke ranging from 0.15 to 0.5% [16]. The defenses are meticulous de-airing, continuous heparinized saline perfusion, aspiration and flushing at every exchange, and strategically uninterrupted therapeutic anticoagulation, which the COMPARE trial showed cuts periprocedural stroke sharply versus bridging [3,15].

### 11.5. Iatrogenic Atrial Septal Defect

Residual septal defects are common after transseptal puncture and are usually benign. An iASD is seen in most patients immediately after the procedure approximately 87% but persists in only about 7% at 12 months [3]. Persistence increases with sheath size: about 20% remain at 1 year after 15F sheaths, while large 21F sheaths used for mitral repair are associated with persistence rates near 50% and worse outcomes [3]. Technique also matters; cryoballoon ablation has been associated with a higher 1-year iASD rate than RF ablation [3]. Most defects require no treatment, whereas large defects causing bidirectional or right-to-left shunting or paradoxical embolism should be closed [3,24].

### 11.6. Other Injuries

Less commonly, the needle injures the coronary sinus or free wall, or puncturing the superior membranous ventricular septum creates a left-ventricular-to-right-atrial communication [3]. Transient arrhythmia at the moment of puncture is usual and self-limited [6]. Detached transseptal-sheath tips are a rare foreign-body complication [3]. Each reinforces the same discipline: confirm position, cross gently, and withdraw the needle the instant the septum yields.

### 11.7. Troubleshooting

Four problems account for most intra-procedural difficulty, and each has a most likely cause and a disciplined response. They are gathered here because they present together at the table even though they belong to different parts of the procedure: a septum that will not tent is a positioning problem answered by the landmark set and the two-directions correction of Section 7; a septum that tents but will not cross is a resistance problem answered by the escalation ladder of Section 10; sudden hypotension is a complication until proven otherwise, and is addressed above; and an unexpected pressure trace is a confirmation problem belonging to Section 9. What unites them is the response they demand: stop, identify the cause, and correct it deliberately rather than by increasing force. Table 6 sets out the four, each with the cause to suspect first and the action that follows.

### 11.8. The Safety Creed

Almost everything that prevents catastrophe in transseptal puncture reduces to a small number of rules. Each has appeared in its own place in the preceding sections, tied to the mechanism that justifies it; gathered, they are short enough to be held in mind during a case and to be taught in a sentence each. They are stated in Table 7 together with the reason behind each, because a rule remembered without its reason is the first to be abandoned under pressure and because every one of them exists to answer a specific way in which the procedure has previously killed patients.

## 12. After Crossing

Should serve as a constant reminder that the puncture is not the end of the transseptal task. Operative steps after crossing carry their own small risks, and maintaining a “routine” keeps them uneventful. Once LA position is confirmed by the routine doctrine, the dilator and then the sheath are advanced into the left atrium, and the needle and dilator are withdrawn, leaving the sheath in the LA [1,2,3]. A guidewire is parked in the left atrium or the left superior pulmonary vein for stability, and the sheath is kept continuously perfused with heparinized saline from this point on [3,15].

Every subsequent catheter exchange through the long sheath is a “fresh opportunity” for clot and air: aspirate to clear any clot, flush, and guard against air at each pass [3]. The residual iatrogenic defect needs no specific action; in most cases, the great majority close over the following year [3].

Accidental withdrawal of the sheath from the LA does not always require a fresh puncture. Guidance back to the puncture site and gradual re-advancement of the steerable catheter through the existing hole may be feasible (initially engaging LA with the in-house guidewire). If that strategy fails, one should follow the initial steps with the dilator (and, if needed, the needle) along the retained track to re-cross the same defect rather than making a new one [3].

## 13. Learning and Competence

Transseptal puncture is operator-dependent, and its safety curve is real and measurable. A common benchmark is that 30 transseptal punctures are needed to pass the steep part of the learning curve [1]. The 2024 EHRA consensus on AF ablation recommends a minimum practical experience of 50 cases [16]. The complication data track this: in one 4690-case series, tamponade was most frequent during an operator’s first 51–100 cases (about 1.3%) and fell to about 0.4% by cases 101–200 [43]. Competence is not a moment but a slope, and it is earned deliberately (Table 8).

Three scaffolds shorten and flatten the curve. Proctoring for the early cases puts an experienced hand in the room when it matters most. Echocardiography is a learning tool as much as a safety one. TEE has been shown to correct a puncture position that fluoroscopy alone placed wrongly in about 16% of cases with inexperienced operators [3], so leaning on it early and weaning toward fluoroscopy-led practice is a reasonable trajectory. Undeniably, simulation and structured training prior to live cases let the mechanics of the pull-down, the two falls, and the confirmation sequence become familiar without a patient on the table. Although electrophysiologists have traditionally trained in the EP laboratory, contemporary precision medicine increasingly supports institutional simulation programs to accelerate learning and reduce complications in complex EP procedures [44,45].

## 14. Conclusions

Seen from first principles, transseptal puncture is striking less for what is new than for what has endured. The anatomy is the same fossa Ross engaged in 1959; the core confirmations pressure, blood, and contrast are his; and today’s flange is simply the original hub indicator in modern form. What has changed is the support around the maneuver: real-time imaging now shows the septum directly, and contemporary devices make difficult crossings more controlled.

The central lesson is procedural discipline: use landmarks to fence the danger zones, require overlapping confirmations before advancing, escalate difficult septa in a structured sequence, and follow a short safety creed that prevents most complications. These steps and the safety gate that governs each of them are collected as a single sequence in Table 9, intended to be read briefly before a case or used as a teaching spine after one. Taught as a reasoned sequence rather than a catheter laboratory trick, transseptal puncture is both learnable and safe.

## Figures and Tables

**Table 1:** Glossary of core terms, placed immediately after the Introduction.

Term	Working Definition
Fossa ovalis	The thin, valve-derived, floor of the interatrial septum the only reliably safe crossing point; the embryological remnant of the septum primum.
Limbus	The muscular rim surrounding the fossa; a raised border the needle must drop over, not through.
True (interatrial) septum	Only the part of the “septum” that separates the two atria without crossing extracardiac space roughly 20% of the apparent septal area.
Anatomic vs. true septum	Much of what looks like septum on imaging is infolded atrial wall (the Waterston/interatrial groove); puncturing there enters extracardiac space, not the left atrium.
Tenting	The pyramidal deformation of the fossa as the needle pushes against it before crossing the visual and tactile signature of correct engagement.
The “pop”/the fall	The sudden loss of resistance as the needle passes into the left atrium; historically described as a “change in resistance”.
The jumps	The one or two downward steps of the sheath tip during pull-down: over the aortic knob/superior vena cava (SVC)–right atrium (RA) junction, then over the limbus into the fossa.
Flange/arrow/indicator/Directional-Hub	The directional marker on the needle hub showing where the tip points present since the first transseptal needles
NCC (non-coronary cusp)	The lowest part of the aortic root; a fluoroscopic marker of the anterior danger boundary, marked classically with an aortic pigtail and, in our center’s workflow, with a retrograde wire.
CS/His landmarks	The coronary sinus (CS) catheter marks the inferior/lateral border of the septum; the His catheter marks the superior border and sits next to the NCC.
iASD	Iatrogenic atrial septal defect the residual hole after sheath removal; common early, mostly closing over a year.
Waterston’s groove	The posterior interatrial groove; a very posterior “puncture” here tracks extracardiac and can bleed late (post sheath removal).

**Table 2:** Historically reported periprocedural heparin regimens and ACT targets.

Source	Heparin	ACT Target
Russo et al. [2]	~2000 U (~¼ dose) before TSP; full 100 U/kg after LA access	250–300 s
Alkhouli et al. [1]	2000–5000 U before; ~200 U/kg total	>300 s (to prevent LA thrombus in PVI)
Di Biase et al./COMPARE [15]	10,000 IU (males)/8000 IU (females) on uninterrupted warfarin, or 15,000 IU bolus off-warfarin, before TSP; infusion thereafter; sheaths pulled at ACT < 200 s	>300 s (on-warfarin)/>350 s (off-warfarin)
Manolis et al. [3]	80–100 U/kg (5000–8000 U) after LA access, then ~2000 U/h	300–350 s
Knecht et al. [12]	50 IU/kg after LA access	not specified
EHRA 2024 consensus (Tzeis et al.) [16]	heparin bolus before transseptal puncture is reasonable, when performed under echocardiographic guidance	At least 300 s

TSP, transseptal puncture; LA, left atrium; PVI, pulmonary vein isolation; ACT, activated clotting time; s, second; U, units; kg, kilo; h, hour; EHRA, European Heart Rhythm Association.

**Table 3:** Device families and representative brands.

Family	Generic Role	Representative Brands
Transseptal needle (mechanical)	Fixed-curve 18 g hollow needle with hub flange	Brockenbrough BRK (~19°)/BRK-1 (~53°); HeartSpan; Cook
RF transseptal needle	Blunt closed tip; crosses by radiofrequency, not force	NRG (Baylis)
Sharp-tip nitinol wire	Crosses via the needle; resumes atraumatic J-shape in LA	SafeSept
Stiff-body pigtail/RF wire	Anti-overshoot exchange rail for large sheaths	ProTrack; VersaCross RF wire
Fixed-curve sheath + dilator	Long sheath through which the system works	Mullins (~8F, 67 cm); Swartz SL0/SL1; Preface; FastCath; Channel
Steerable sheath	Deflectable long sheath	Agilis; TorFlex
Guidewire	SVC access/exchange rail	J-wire 0.032–0.035″ (some sheaths accept < 0.032″)
Pressure transducer	LA/RA waveform confirmation	Standard fluid-filled pressure line

RF, radiofrequency; LA, left atrium; SVC, superior vena cava; RA, right atrium.

**Table 4:** Fluoroscopy vs. TEE vs. ICE: an objective, preference-free comparison.

Parameter	Fluoroscopy	TEE	ICE
Direct septal imaging	No (inferred)	Yes	Yes
Puncture-height resolution	Limited	Yes (bicaval/4-chamber)	Yes
Site-specific targeting	Approximate	Yes	Yes
Value in difficult/failed cases	Lower	Higher	Higher
Anesthesia	Local/conscious	General or deep sedation	Local/conscious
Extra venous access	No	No	Yes
Extra operator	No	Yes (echocardiographer)	Usually operator-run
Relative cost	Low	Higher	Highest (disposable catheter)
Patient convenience	High	Lower (probe/GA)	Moderate

TEE, transesophageal echocardiogram; ICE, Intracardiac echocardiography; GA, general anesthesia.

**Table 5:** Summary of enabling technologies (beyond modified needles) in the setting of transseptal puncture.

Technology	Mechanism	Mechanical Force Needed	Sheath Exchange Needed	Advantage	Evidence
Direct large sheath access	large bore sheath for ablation catheter delivery used also for TSP	yes	no	shorter TSP time Low or zero fluoroscopy	nonrandomized cohorts
Electrified J wire	electrocautery pen tip placed in contact with the proximal portion of the guidewire	no	no	shorter TSP time fluoroless	randomized evidence
3D-EAM	EP based criteria such as unipolar current-of-injury amplitude	yes	yes	fluoroless	nonrandomized cohort
Rotational probing technique	Rotation without needle advancement	yes	yes	inexpensive, no workflow modification	Nonrandomized cohort

3D-EAM, three-dimensional electroanatomic mapping; EP, electrophysiology; RF, radiofrequency; TSP, transseptal puncture.

**Table 6:** Troubleshooting the four classic intra-procedural problems.

Problem	Most Likely Cause	Action
**Will not tent/no engagement**	Tip not in the fossa too superior or anterior, or riding on the limbus rather than dropping into it	Re-map CS/His/NCC landmarks; apply the “two-directions” correction (retract + rotate together); reshape the needle; drop lower from the SVC
**Tents but will not cross**	Fibrotic, thick, or scarred (often prior-TSP) resistant septum	Climb the escalation ladder (stylet → sharp nitinol wire → RF); consider deep-inspiration maneuver; do not apply reckless force (lurch risk)
**Sudden hypotension**	Hemopericardium/tamponade until proven otherwise	Urgent echocardiography; open pericardiocentesis tray; reverse heparin (protamine ± FFP); surgical backup; do not attribute to vagotonia
**Unexpected pressure trace**	Arterial trace = aortic puncture; dampened/absent with tenting = non-penetration; absent without tenting = needle-lumen clot	If arterial: do NOT advance, withdraw needle, observe for tamponade. If non-penetration: reconfirm fossa position and re-engage If clot: withdraw, clear with stylet/heparinized flush

CS: coronary sinus; NCC: non-coronary cusp; SVC: superior vena cava; RF: radiofrequency; TSP: transseptal puncture; FFP: fresh frozen plasma.

**Table 7:** The safety creed: the small set of rules that prevent most catastrophes.

Rule	Why
**Confirm before every advance**	A single confirmation can mislead; overlap them
**Never dilate a tract you have not confirmed is in the left atrium**	Dilating a wrong tract turns a needle-stick into a catastrophe
**If the sheath is in the aorta, do not withdraw it**	Withdrawal can cause fatal bleeding; leave it, call surgery
**Aspirating air means you are extracardiac**	Air returns from bronchial tree or esophagus, not from the left atrium
**Hypotension is hemopericardium until proven otherwise**	Vagotonia is the rarer explanation; treat the dangerous one first
**The instant the septum yields, withdraw the needle tip**	Prevents the forward lurch into the posterior wall
**When in doubt, withdraw and restart**	Pushing harder converts doubt into perforation

**Table 8:** The first fifty cases: what to expect, and what to do.

Phase	What to Expect	What to Do
**Cases 1–10**	Highest uncertainty; every step feels deliberate	Work proctored; keep echo as backup; run the full confirmation doctrine unhurried; low threshold to pause and reassess
**Cases 10–30**	Pattern recognition forming; still on the steep curve	Never abbreviate the confirmations; log every case and any complication; drill the “two-directions” RAO correction
**Cases 30–50**	Competence emerging; complication risk falling	Begin supervised difficult-septum exposure; move toward fluoroscopy-led practice where appropriate; build the escalation ladder into reflex
**Throughout**	Complacency is the new hazard	Never skip confirmation; treat hypotension as tamponade; always alert to tackle complications

**Table 9:** The whole procedure (based on our institutional workflow) at a glance, each step with its governing safety gate.

Step	Action	Safety Gate
**1 Prepare**	Ultrasound-guided femoral venous access ± arterial line; TEE thrombus screen; anticoagulation plan; assemble, flush and de-air the system	Pre-flight checklist complete
**2 Set landmarks**	Coronary sinus catheter; His-surrogate; non-coronary-cusp wire; spine	Safe zone fenced on all sides
**3 Pull down (AP)**	Retract as a single unit, flange ~3 o’clock; watch for the two falls	Second, subtle fall = the fossa
**4 Orient**	RAO (anterior–posterior) → lateral (aortic clearance) → LAO ≈ 45°	Tip posterior, clear of the aorta
**5 Engage**	Rotate to 4–5 o’clock (target-specific); tent the fossa; stain with contrast	Contrast holds in the fossa
**6 Confirm**	Pressure + aspiration + contrast direction (+ PV wire as needed)	All signs concordant before anything advances
**7 Cross**	Advance the needle; withdraw the tip the instant the septum yields	No forward lurch
**8 Advance**	Dilator (dilate the tract) then sheath into the LA	Never dilate an unconfirmed tract
**9 After**	Withdraw needle/dilator; perfuse the sheath; aspirate and de-air every exchange	Hypotension = tamponade until proven otherwise

TEE, transesophageal echocardiography; RAO, right anterior oblique; LAO, left anterior oblique; LA, left atrium.

## Data Availability

No new data were created or analyzed in this study.
